# Special Issue on Versatile Organ-on-a-Chip Devices

**DOI:** 10.3390/mi12121444

**Published:** 2021-11-25

**Authors:** András Dér

**Affiliations:** Eötvös Loránd Research Network (ELKH) Biological Research Centre, 6726 Szeged, Hungary; der.andras@brc.hu

The tremendous success of microelectronics at the end of the 20th century, often symbolized by “Moore’s law”, is based on miniaturization of the active and passive elements of electronic circuits. Improving the methods of the underlying micro- and nanofabrication technique, lithography actually paved the way for the development of various “lab-on-a-chip” (LOC) applications, which complement microelectronics by miniature fluidic, mechanical, and/or optical elements. Since biological cells and their 2D or 3D cultures, organoids (miniature organ models), can be easily accommodated by LOC structures, it was only a question of time when this trend reached biological and medical science. Miniature organ models of brain, heart, lung, muscle, blood vessels, liver, and skin have been created during the past 10 years to model and study the cellular and molecular interactions and pathological conditions of these organs. Predictably, such “organs-on-a-chip” will increasingly represent a cornerstone of translational medicine in the forthcoming decades, and may complement, or in many cases substitute, animal studies in modeling diseases. Given the complexity of the human body, however, such disease models should also include the interaction of organs with each other (e.g., gut epithelium, microbiome, blood-brain barrier, brain for the “gut-brain axis”, etc.). The goal of this Special Issue was to facilitate the solution of this task by giving an overview of various existing approaches for versatile organ-on-a-chip models, and stimulating cooperation among experts of different areas.

We collected six regular research papers and two review articles that focus on novel methodological developments related to various aspects of organ-on-a-chip technologies, and their utilizations in basic or applied sciences.

The papers can be classified according to different aspects. In terms of the area of interest, most of the articles aim at the physiological characterization of biological barriers of endothelial or epithelial origin [1,2,3,4] and other organs or tumors [5,6,7].

According the requirements of the particular application, various chip geometries and structure-building strategies are discussed, allowing studies of the characteristically two-dimensional barrier layers, or even 3-D organoids at different levels of complexity and miniaturization [4,5,6].

Working with living biological cells and tissues requires a special expertise in material engineering as well. Some papers discuss biocompatibility issues of the building blocks of the developed biochips [1,4,6], while others deal with the optimization of the material properties utilized (e.g., in drug delivery applications by microparticles [8], or modeling tumor microenvironment [5]).

A key feature of organ-on-a-chip devices is the versatility they offer for measuring and testing strategies. Besides the opportunity for microscopic observation, which is a general feature of the various chips presented in this Special Issue, two of the papers focus on the electric properties of quasi-2D tissues, by the application of trans-endothelial electric resistance and microelectrode ion flux measurements ([3] and [1], respectively). The importance of other physical and physico-chemical parameters, such as shear stress and permeability are also emphasized throughout the papers [4,6]. The majority of the papers presents a detailed theoretical analysis [2,4,7] or even computer modeling [3,5], as well.

In the order of publication dates of the research papers, Buchroitner et al. designed a new type of insert for transwell measurements, and fabricated it with high-resolution photopolymerization [1]. The novel system allows an investigation of physiological processes in direct cell-to-cell co-culture by simultaneous ion-flux (MIFE) and fluorescence microscopy measurements.

Acosta-Cuevas et al. deal with optimization strategies of photopolymerized microparticles, created from polyethylene glycol diacrylate (PEGDA) by using microfluidic devices, for various applications [8]. The emphasis was on the effects of cross-linker concentration and initial gelation time on the structural and mechanical properties of the microparticles (e.g., cross-linking density, compressibility, swelling, drying, soluble fraction), and practical applications in encapsulation of microorganisms, or release of medicines are envisaged.

Ma et al. developed simple and cost-effective techniques to build laminated and stacked structures, utilizing a biocompatible double-sided adhesive to form single, straight microchannels [4]. The microchips are especially well-suited for studies where the determination of shear-stress dependence of some crucial properties of endothelial or epithelial layers in a large dynamic range is required.

Bojin et al. present tissue constructs fabricated by 3D-bioprinting as models of tumor microenvironment (TME) [5]. The results confirm the hypothesis that scaffold-based models of the TME can be fabricated by extrusion bioprinting. By the aid of computer simulations, they suggest that the formation of experimentally observed aggregates comprising malignant cells and peritumoral cells can be explained by the differential adhesion hypothesis. Engineered TME models are promising tools for both fundamental research and medical applications, where personalized anti-cancer therapies can be tested on patient-specific tumor organoids.

Goldstein et al. present two 3D-print-based fabrication methods for a generic multi-organ-on-a-chip device, one with a PDMS microfluidic core unit and one based on 3D-printed units [6]. The device was designed to allow tissue crosstalk, using microchannel configuration and permeable membranes. The authors used computational fluid dynamics simulations to show that significant differences in shear stress can be created between individual culture compartments. They offer a cost-effective, accessible protocol enabling the design and fabrication of advanced multi-organ-on-a-chip devices.

Nasiri et al. used computer simulations to design (and a soft-lithography technique to fabricate) a hybrid inertial and magnetophoretic microfluidic device for the separation of circulating tumor cells (CTCs) from blood [7]. They established that at an optimal flow rate, the efficiencies of both the recovery rate and the purity were maximal (95% and 93%, respectively). They suggest the utilization of their chip device for precise, fast, and specific separation of various target cells with a high efficiency.

The Special Issue is concluded by two gap-filling review papers. Ponmozhi et al. provide an overview of the state-of-the-art skin-on-a-chip technologies and devices [2]. First, the diffusion materials are summarized (membranes, excised human and animal skins and reconstructed skin tissues), then various miniaturized platforms are presented. In the next session, the application possibilities are summarized, including active ingredient diffusion and toxicological studies, pharmacological and wound healing experiments, cosmetological investigations on skin repair, inflammation, ageing, and shear stress. At the end, the main problems to be solved and the outlook are discussed.

Vígh et al. present a critical review of the various methods used to characterize the trans-endothelial and epithelial resistivity (TEER), with special respect to the blood-brain barrier (BBB) [3]. TEER is a crucial physical parameter to characterize the barrier tightness of the in vitro BBB models. They describe the setups, electrodes, and instruments that are used to measure TEER across brain microvessels and in culture models of the BBB, as well as critically assess the influence of often neglected physical and technical parameters (e.g., temperature, viscosity, current density generated by different electrode types, surface size, circumference, and porosity of the culture insert membrane). The review is expected to be a reference work for the electrical characterization of BBB (and other biological barriers, as well).

I believe that this selected set of papers can provide a good teaser about the complexity of the rapidly developing field of organ-on-a-chip devices, and that it can serve as a reference for a number of future research projects.

Last but not least, I would like to take this opportunity to thank all the authors for publishing their noteworthy scientific results in this Special Issue, and the reviewers for dedicating their time to improve the quality of the submitted papers. My special thanks to the Section Managing Editor, Mr. Jerry Chen, for his continuous support in the management and promotion of this Special Issue, as well as to my co-Guest Editors, Drs. Fruzsina Walter, Sándor Valkai and András Kincses for their valuable help throughout the editorial process. Many thanks also to Mr. Dániel Petrovszki for his technical help.
